# Editorial Comment from Dr Sakamoto to Solitary recurrence of prostate cancer surrounded by seminal vesicle/vas deferens‐like epithelium

**DOI:** 10.1002/iju5.12177

**Published:** 2020-08-01

**Authors:** Shinichi Sakamoto

**Affiliations:** ^1^ Department of Urology Chiba University Graduate School of Medicine Chiba Japan

Metastasis of prostate cancer predominantly takes place in bone and regional lymph nodes. However, metastases to uncommon regions are often regarded as a poor prognostic sign.^1^, ^2^

Takamori *et al*. reported a very rare case of prostate cancer recurrence that seemed to be surrounded by seminal vesicle/vas deferens‐like epithelium after radiation therapy.^3^

Based on the previous report, congenital cystic anomalies of the seminal vesicle may be classified in to three groups, such as (i) isolated cyst, (ii) cysts associated with upper urinary tract anomalies, and (iii) cysts associated with autosomal dominant polycystic kidney disease. During development, cysts of the seminal vesicle emerge when one is in their 20s and become sexually active. Seminal vesicle cysts are usually below 5 cm, which is often discovered by various symptoms, such as urinary tract infections, infertility, prostatitis, hematuria, and hemospermia.^4^, ^5^ Cyst of the seminal vesicle is often associated with ipsilateral renal agenesis in around 60% of the patients. The reason seems that the development of the seminal vesicle cyst associated with the defect in the development of the distal mesonephric duct and misdeveloped ureteral budding, which results in the renal agenesis.^4^, ^5^

Regarding the ectopic prostate, although it is rare, there were several reports in the past. The most common site seems to be the bladder and urethra, followed by seminal vesicle, testis, and epididymis. Some reports also indicated the presence of ectopic prostate in the cervix and vagina in females.^6^ The mechanism for the emergence of ectopic prostate has been related to the migration and misplacement of normal tissue, metaplastic change due to chronic inflammation, and retained embryonic tissue.

In the current case, neither renal agenesis nor any symptoms existed.

So, the chance of existence of a seminal vesicle cyst before the emergence of recurrence seems to be low.

Another possibility may be related to the ectopic prostate that developed to be a malignant cancer with the seminal vesicle/vas deferens‐like epithelium.

Due to limited previous evidence, we seem to have no clear answer. However, this case will bring about new insights into the metastatic feature of prostate cancer.

Since metastasis with seminal vesicle‐like epithelium is rare, the long‐term prognosis of this case may be of interest.

## Conflict of interest

The author declares no conflict of interest.

## References

[1] Yamada Y , Sakamoto S , Rii J *et al* How many bone metastases may be defined as high‐volume metastatic prostate cancer in Asians: a retrospective multicenter cohort study. Prostate 2020; 80: 432–40. 32017175 10.1002/pros.23958

[2] Sakamoto S , Maimaiti M , Xu M *et al* Higher serum testosterone levels associated with favorable prognosis in enzalutamide‐ and abiraterone‐treated castration‐resistant prostate cancer. J. Clin. Med. 2019; 8: 489. 10.3390/jcm8040489 PMC6518240 30978937

[3] Takamori H , Kamba T , Sumiyoshi S *et al* Solitary recurrence of prostate cancer surrounded by seminal vesicle/vas deferens‐like epithelium. IJU Case Rep. 2020; 3: 171–3. 10.1002/iju5.12168 PMC7469812 32914063

[4] Arora SS , Breiman RS , Webb EM , Westphalen AC , Yeh BM , Coakley FV . CT and MRI of congenital anomalies of the seminal vesicles. Am. J. Roentgenol. 2007; 189: 130–5. 17579162 10.2214/AJR.06.1345

[5] Gorrea M , Lorente R , Roel J . Seminal vesicle cyst associated with ipsilateral renal agenesis and papillary carcinoma of the bladder. Eur. Radiol. 2001; 11: 2500–3. 11734948 10.1007/s003300000815

[6] Tan FQ , Xu X , Shen BH *et al* An unusual case of retrovesical ectopic prostate tissue accompanied by primary prostate cancer. World J. Surg. Oncol. 2012; 10: 186. 22966979 10.1186/1477-7819-10-186 PMC3556091

